# Radiation treatment of hemato-oncological patients in times of the COVID-19 pandemic: Expert recommendations from the radiation oncology panels of the German Hodgkin Study Group and the German Lymphoma Alliance

**DOI:** 10.1007/s00066-020-01705-w

**Published:** 2020-10-30

**Authors:** M. Oertel, K. Elsayad, R. Engenhart-Cabillic, G. Reinartz, C. Baues, H. Schmidberger, D. Vordermark, S. Marnitz, P. Lukas, C. Ruebe, A. Engert, G. Lenz, H. T. Eich

**Affiliations:** 1 Department of Radiation Oncology, University Hospital Muenster, Albert-Schweitzer-Campus 1 building A1, 48149 Muenster, Germany; 2 Department of Radiotherapy and Radiation Oncology, University Hospital Giessen-Marburg, Marburg, Germany; 3 Department of Radiation Oncology and Cyberknife Center, University Hospital of Cologne, Cologne, Germany; 4 Department of Radiotherapy and Radiation Oncology, University Hospital Mainz, Mainz, Germany; 5 Department of Radiation Oncology, University Hospital Halle (Saale), Halle (Saale), Germany; 6 Department of Radiooncology, Medical University Innsbruck, Innsbruck, Austria; 7 Department of Radiation Oncology, Saarland University Hospital, Homburg, Germany; 8 Department I of Internal Medicine, Center for Integrated Oncology Aachen Bonn Cologne Düsseldorf, University Hospital of Cologne, Cologne, Germany; 9 Department of Medicine A, Hematology, Oncology, University Hospital Muenster, Muenster, Germany

**Keywords:** COVID, SARS-CoV-2, recommendation, lymphoma, hematology

## Abstract

**Purpose:**

The coronavirus pandemic is affecting global health systems, endangering daily patient care. Hemato-oncological patients are particularly vulnerable to infection, requiring decisive recommendations on treatment and triage. The aim of this survey amongst experts on radiation therapy (RT) for lymphoma and leukemia is to delineate typical clinical scenarios and to provide counsel for high-quality care.

**Methods:**

A multi-item questionnaire containing multiple-choice and free-text questions was developed in a peer-reviewed process and sent to members of the radiation oncology panels of the German Hodgkin Study Group and the German Lymphoma Alliance. Answers were assessed online and analyzed centrally.

**Results:**

Omission of RT was only considered in a minority of cases if alternative treatment options were available. Hypofractionated regimens and reduced dosages may be used for indolent lymphoma and fractures due to multiple myeloma. Overall, there was a tendency to shorten RT rather than to postpone or omit it. Even in case of critical resource shortage, panelists agreed to start emergency RT for typical indications (intracranial pressure, spinal compression, superior vena cava syndrome) within 24 h. Possible criteria to consider for patient triage are the availability of (systemic) options, the underlying disease dynamic, and the treatment rationale (curative/palliative).

**Conclusion:**

RT for hemato-oncological patients receives high-priority and should be maintained even in later stages of the pandemic. Hypofractionation and shortened treatment schedules are feasible options for well-defined constellations, but have to be discussed in the clinical context.

**Electronic supplementary material:**

The online version of this article (10.1007/s00066-020-01705-w) contains supplementary material, which is available to authorized users.

## Introduction

In December 2019, the first cases of an enigmatic pneumonia emerged in the city of Wuhan, Hubei Province, China. The causative virus was later identified as a member of the RNA-containing family of *Coronaviridae*, named severe acute respiratory syndrome coronavirus 2 (SARS-CoV‑2), with the resulting disease being called coronavirus disease 2019 (COVID-19) [1]. Clinical presentation shows great heterogeneity, from asymptomatic courses to typical pneumonia symptoms like fever, cough, and dyspnea, but also includes nausea, diarrhea, and kidney injury, which may require intensive care [2–4]. Particularly high fatality rates are seen amongst patients receiving mechanical ventilation due to respiratory failure [2–4]. At the time of submission of this article, 31.1 million persons worldwide had been infected, of whom 962,000 had succumbed to the disease [5].

Oncological patients may be particularly prone to infection, as Chinese authors claim an increase of infection rate, severe courses, and fatality rates for oncological patients, with the latter reaching up to 28.6% [6–9]. Focusing on hematological malignancies, reports from Europe and the United States displayed case-fatality rates of up to 40% [10–12].

Data from New York City reveal an increased rate of intubation amongst cancer patients with COVID-19, although augmented mortality was limited to patients <50 years of age [13]. Against this background, infection risks have to be weighed against the possible disadvantage of postponed or omitted therapy [14, 15].

Although the exact impact of COVID-19 on cancer patients is yet to be determined, many specialists in radiation oncology have already formulated emergency guidelines and recommendations for treatment of oncological patients during the pandemic [15–21]. Regarding hemato-oncological patients, the recent consensus paper by the International Lymphoma Radiation Oncology Group (ILROG) has outlined strategies for various entities. Still, there is an unmet need for treatment recommendations, which prompted us to form an expert panel consisting of the main radiation oncologists of the German Hodgkin Study Group (GHSG) and the German Lymphoma Alliance (GLA). The hereby presented paper analyzes typical scenarios and aims at providing guidance for clinical practice.

## Methods

A questionnaire was developed by the radiation therapy (RT) expert panel in a peer-reviewed process and circulated within the group in May 2020 (Table 1 for clinical cases and supplementary Fig. 1 for full questionnaire). Recipients were either heads of departments of radiation oncology or consultants specialized in the field of hematological malignancies within the GHSG and GLA. Two radiation oncology departments did not answer the questionnaire. Answers were due on May 15, 2020. The survey is divided into two parts representing consecutive phases of the COVID-19 pandemic. The first scenario (phase 1) describes an early situation during the pandemic in which sufficient personal and treatment capacities are available, although meanwhile, the disease is spreading and thereby endangering patient care. Later, the second phase (phase 2) is characterized by a critical shortage in resources, which might require a triage concerning patient care. The survey was conducted as an online interrogation with a local adaption of the Lime survey (Lime Survey, Hamburg, Germany). Data were analyzed with Microsoft Excel 2016 (Microsoft Corporation, Redmond, USA).

**Table 1:** Overview of the clinical scenarios for which different questions concerning priority, delay, and omission of therapy had to be answered

Case 1:	Painful osteolytic lesion caused by multiple myeloma in non-weightbearing bones after stabilizing surgery
Case 2:	Osteolytic lesion of multiple myeloma in weightbearing bones (e.g., axial skeleton) without surgery
Case 3:	Limited-stage Hodgkin lymphoma, Ann–Arbor stage II without risk factors after completion of two cycles of ABVD
Case 4, 5:	Diffuse large B‑cell lymphoma with initial abdominal bulky disease after completion of six cycles of R‑CHOP 4) With no information on PET status 5) PET-positive after treatment
Case 6:	Early-stage indolent lymphoma in noncritical location

*ABVD* adriamycin, bleomycin, vinblastine, dacarbazine; *R-CHOP* rituximab, cyclophosphamide, hydroxydaunorubicin, oncovin, prednisone; *PET* positron emission tomography

## Results

Overall, 10 participants answered the questionnaire. Most departments have taken protective measures for hemato-oncological RT patients (9; 90%), including the wearing of face masks by patients and staff and rigid basic hygiene (disinfection of treatment rooms, social distancing, reduction of waiting time) (Table 2). Additionally, screening with a decisive questionnaire is implemented in most departments in order to identify possible infectious patients. Tests for COVID-19 infection are widely applied, but to different populations (all patients, all in-patients, all symptomatic patients, all staff, all symptomatic staff).

**Table 2:** Overview of answers provided for general questions (*n* = 10)

	Yes	No	Not answered/indecisive
Hygiene measures taken	9	1	0
*Phase 1*			
Reduction of hemato-oncological patients	0	10	0
Critical indication for thoracic RT	3	6	1
CT during follow-up	4	4	2
Reduction of steroids	3	6	1
*Phase 2*			
Reduction of hemato-oncological patients	3	4	3
Chemotherapy-only for early-stage HL	2	5	3
Omission of TBI as conditioning regimen before allogenic SCT	3	2	5

*RT* radiotherapy, *CT* computed tomography, *HL* Hodgkin lymphoma, *TBI* total body irradiation, *SCT* stem cell transplantation

Participants overwhelmingly declined to reduce hemato-oncological patient numbers in both phase 1 (10/10) and 2 (4/7). Three participants recommended a more critical indication for thoracic RT, whereas 6 denied this. For corticosteroids as an immunosuppressive agent, only a minority of participants considered a reduction (3/10). To facilitate differential diagnosis between viral pneumonia and radiation pneumonitis, 50% of participants advocated the use of a computed tomography (CT) scan (4 vs. 4).

### Phase 1

**Fig. 1:**
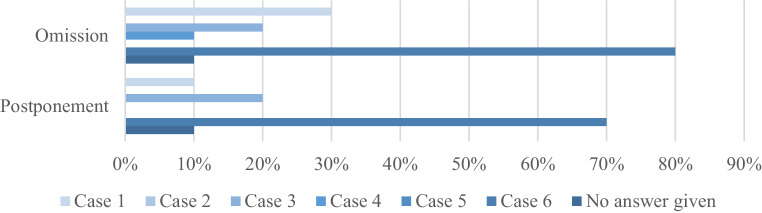
Overview of answers concerning postponement and omission of radiotherapy as provided by the participants for clinical cases in phase 1 of the pandemic (*n* = 10)

With multiple answers possible, there was a clear denial to delay RT in this phase of the pandemic, except for case 6 (7; 70%), case 3 (2; 20%), and case 1 (1; 10%; Fig. 1). The suggested time to postpone treatment was 4 weeks for case 1 and 3, 4–12 weeks of delay were proposed for case 6. Some answers argue for a wait-and-see strategy in this latter case, with a limitation of treatment to symptomatic/progressive disease. Omission of RT in case of resource shortage was considered for scenarios 6 (8; 80%), 1 (3; 30%), 3 (2; 20%), and 4 (1; 10%).

Shortening of treatment via hypofractionation or limitation of total dose was discussed for nearly all examples given with various dose prescriptions. There were suggestions to use hypofractionation in case 1 (5; 50%) with 1*8 Gy, 5*4 Gy, 8*3 Gy, and 10*3 Gy; case 2 (5; 50%) with 5*4 Gy and 8–12*3 Gy; case 4 (1; 10%) with 9–10*3 Gy; case 5 (1; 10%) with 12*3 Gy; and case 6 (2; 20%) with 1*4 Gy and 9*3 Gy. Dose-reduced concepts were proposed especially for scenario 6 (7; 70%), the majority of suggestions being 2*2 Gy.

In case of a COVID-19 infection before the onset of treatment, most participants agreed to postpone treatment for cases 6 (8; 80%), 1 (6; 60%), and 4 (5; 50%), with less than 50% approval for postponing treatment for the remaining three other cases. Similar cases were found to be eligible for interruption of treatment until COVID-19-negativity is established (case 1 and 6: 60%, respectively).

### Phase 2

In contrast to phase 1, considerations for reduction of patients increased (3; 30% “yes” versus 4; 40% “no”; Table 2). Factors for clinical triage are largely disease dependent, for example the onset of symptoms, curative versus palliative treatment concept (7; 70% each), and the availability of alternative (systemic) treatments (6; 60%), whereas patient-related risk factors (smoking, hypertension, diabetes, age) were of less importance (4; 40%). The patient’s immune status, immunosuppressive potency of therapy, and the possibility to carry out treatment in an outpatient setting were deemed even less essential (3; 30% each).

**Fig. 2:**
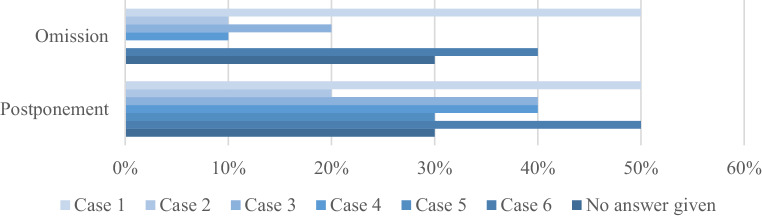
Overview of answers concerning postponement and omission of radiotherapy as provided by the participants for clinical cases in phase 2 of the pandemic (*n* = 10)

There was a broad consensus to commence emergency RT within 24 h for most given examples (7; 70% for superior vena cava syndrome, spinal cord compression, and amaurosis due to intraorbital disease; 6; 60% for intracranial pressure due to central nervous system lymphoma), except for the case of pain caused by vertebral involvement (2; 20%). No agreement was achieved concerning the omission of time-intense RT treatments like total body irradiation (TBI; 3; 30% in favor vs. 2; 20% against with 5; 50% being indecisive). Regarding the presented clinical situations, omission of RT due to limited capacities was considered for cases 1 (5; 50%) and 6 (4; 40%), with small percentages for the other scenarios (Fig. 2). Heterogeneous answers were received with regard to the question of which patients to postpone, focusing on cases 1 and 6 (5; 50% each) as well as cases 3 and 4 (4; 40% each). Additionally, the accepted delay until the beginning of RT differed significantly, being days up to 4 weeks for case 1, 4–6 weeks for case 2, 2–6 weeks for case 3, 3–8 weeks for case 4, 3–4 weeks for case 5, and 8–12 weeks for case 6.

Focusing on clinical case 3 (Hodgkin lymphoma), most participants disagreed on the idea of a chemotherapy-only regimen (5; 50% against vs. 2; 20% in favor). Ranking the different scenarios showed significant differences regarding urgency, with the highest priority for case 2 (4; 40%) and the lowest priority for case 6 (6; 60%). Median ranks, taking into account only complete answers, were: 4, 1, 2.5, 3, 2, and 6 for cases 1–6, respectively, which defines scenario 2 and 5 as the most prioritized cases.

## Discussion

The hereby presented survey mirrors the complexity of hemato-oncological patient care in radiation oncology facing a pandemic. It emphasizes the importance for RT treatment for most clinical scenarios and illustrates feasible alternative concepts.

Cancer patients are defined as one group of persons at risk for a severe course of COVID-19 infection [22]. They rely on consequent basic hygiene and social distancing, which have been implemented in nearly all departments for patient and staff protection.

The immunosuppressive state of many oncological patients renders them susceptible to infection with increased rates of morbidity and mortality. Reports from China describe a doubled risk for COVID-19 infection and fatal course of disease for cancer patients [7, 8]. In accordance with this finding, a retrospective analysis of 28 cancer patients with COVID-19 identified anti-cancer treatment within the last 14 days as a risk factor for severe events (only one patient received RT in this group) [9]. Although RT has traditionally been regarded as an immunosuppressive treatment, recent studies in radiobiology rather suggest a complex immunomodulatory role, acting on both the cellular and humoral level of the immune system [23, 24].

Due to the increased risk profile of hemato-oncological patients, it may be attractive to increase screening efforts and to avoid supplementary toxicities. Nevertheless, there was no consent to decrease thoracic irradiations to prevent putative cardiopulmonary side effects. In accordance with this result, a decreased use of corticosteroids was not approved. A recent review from Guy’s Hospital failed to determine the use of steroids as a risk factor for COVID-19 patients and even pointed towards a positive role when used in early stages of infection [25]. The idea of CT scans as a means of differentiation between radiation pneumonitis and COVID pneumonia evoked mixed responses. Importantly, CT signs of COVID-19, like ground-glass opacities and consolidations, are unspecific and may also be caused by influenza or can be confused with radiation pneumonitis [26–28]. Consequently, a medical indication is needed, as thoracic CT scans neither replace laboratory tests nor should they be used as a screening method [26–28].

Recently, the ILROG published “emergency” guidelines for the treatment of leukemia and lymphoma patients in times of a pandemic, which define three key strategies: omission, delay, or shortening of RT [15].

Abandonment of RT may be possible for a palliative setting in which alternative options are at hand, as indicated in clinical scenario 1. Consequently, half of the participants agreed to omit or postpone treatment by up to 4 weeks for a comparable patient in a later phase of the pandemic. Additionally, 60% of answers suggest a delay or interruption of treatment in this case if the patient tests positive for SARS-CoV‑2. Importantly, most participants recommend shortening rather than omission of RT, especially in phase 1 of the pandemic. Regarding multiple myeloma, there were various concepts ranging from 1*8 Gy, over 5*4 Gy to 10–12*3 Gy. Although hypofractionation is feasible, the respective concepts have to be considered within the clinical context and adapted to the individual patient. Rades et al. demonstrated superior neurological recovery for myeloma patients with spinal cord compression by using RT doses of ≥30 Gy [29]. Additionally, solitary plasma cell lesions such as single osseous plasmocytoma or extramedullary plasmocytoma may benefit from dose escalation, with a dose recommendation of 40–50 Gy provided by the ILROG [30–32].

The questionnaire also aimed at judging the use of a chemotherapy-only regimen for early-stage Hodgkin lymphoma (case 3), which has been addressed by three multicenter trials. Noninferiority of unimodal treatment could not be proven, so combined-modality treatment of 2–3 cycles of ABVD followed by involved-site or involved-node RT remains standard of care for early-stage patients [33–35]. This corresponds to the low percentage of panelists who agreed to this chemotherapy-only strategy.

De-escalation of RT doses for lymphoma patients has been of interest in recent years, especially for indolent lymphoma [36, 37]. The FORT trial demonstrated feasibility and efficacy of 2*2 Gy in comparison to 24 Gy. Although the low-dose regimen showed inferior progression-free survival and local control, overall survival did not differ significantly [36]. Further studies elaborated on the feasibility of this concept for extranodal indolent lymphoma [38–41]. This rationale encouraged the suggestion of 2*2 Gy as an alternative treatment strategy for scenario 6. Other options may be the postponement of RT for 1 up to 3 months, which reached a high consent, or a wait-and-see strategy till the onset of symptoms.

This is contrasted by the aggressive biology of diffuse large B‑cell lymphoma described in cases 4 and 5 [42]. Delay in treatment delivery for these patients is only acceptable when facing a critical shortage of resources. Furthermore, hypofractionated concepts lack study validation, being based mainly on calculated equivalent biological efficacy [15].

Overall, treatment of leukemia and lymphoma patients is prioritized and shortage of treatment for these patients is neglected in most situations. This is in accordance with the agreement to start emergency radiation treatments for nearly all provided indications, even during the later phase of the pandemic. Moreover, TBI, as an established conditioning modality before allogenic stem cell transplantation, should not be discarded even in advanced pandemic stages [43].

However, adequate triage remains challenging both at the medical and the ethical level, being a multifactorial process. Panel members consented to integrate the distinction between curative and palliative regimens, onset of symptoms/biology of the underlying disease, and the availability of (systemic) alternatives in the decision process. It is the authors’ strong belief that access to high-quality RT has to be sustained for all patients, while maintaining treatment equality and personal dignity as delineated by the German federal ethic commission [44].

This survey bears several limitations, as only a limited number of radiation oncology experts in lymphoma treatment were consulted. Although the inclusion of different institutions aims at reflecting various concepts, this may not reveal the complete picture. Additionally, nearly all participants work at university hospitals, thus giving a one-sided view of treatment. Restriction of the survey to predefined scenarios facilitated the evaluation process, but may be prone to oversimplification of the complex clinical reality.

Nevertheless, this article provides concepts for typical indications for RT in hemato-oncological patients which are of interest for everyday clinical practice. The hereby provided recommendations are of value until the end of the current pandemic and may serve as a blueprint for future epidemics or crises. Altogether, treating physicians should focus on both maintaining public health by limiting virus spread within the population and guaranteeing state-of-the-art hemato-oncological cancer care.

## Conclusion

COVID-19 will most likely remain a challenge for health systems at the national and international level in the months to come. RT for hemato-oncological patients receives high priority and should be maintained even in later stages of the pandemic. Hypofractionation and shortened treatment schedules are feasible options for well-defined constellations, but have to be discussed in the clinical context.

## Caption Electronic Supplementary Material

Supp. Fig. 1 Full questionnaire as answered by the participants. Questions were assessed consecutively
