# Supplementary material for: Radiation treatment of hemato-oncological patients in times of the COVID-19 pandemic: Expert recommendations from the radiation oncology panels of the German Hodgkin Study Group and the German Lymphoma Alliance
Source: Strahlenther Onkol. 2020 Oct 30;196(12):1096–102. doi: 10.1007/s00066-020-01705-w (PMC7596809; doi:10.1007/s00066-020-01705-w)
Supplement: Supplementary file 1 — Supp. Fig. 1 Full questionnaire as answered by the participants. Questions were assessed consecutively [file 66_2020_1705_MOESM1_ESM.docx]

**First phase of the pandemic**

In the first phase of the pandemic sufficient resources are available to enable adequate patient care. Nevertheless, the spread of infection, affecting staff and/or patients, may potentially endanger treatment

1. *Do you consider reducing the number of hemato-oncological patients in your radiation oncology department?*

- Yes
- No

1. *Do you take special precautions for the protection of hemato-oncological patients?*

- Yes
- No

1. *If yes, which measures?*
2. *Is there a critical consideration of the indication for thoracic radiotherapy (RT) concerning its potential cardiopulmonary side effects?*

- Yes
- No

1. *Would you reduce the use of steroids during RT to avoid potential immunosuppression?*

- Avoid
- Reduce
- No changes

1. *Would you use a low-dose CT for follow-up to enable differential diagnosis between radiogenic pneumonitis and atypical pneumonia as caused by SARS-CoV-2?*

- Yes
- No

**Clinical cases (questions 7-12)**

Case 1: Painful osteolytic lesion caused by multiple myeloma in non-weightbearing bones after stabilizing surgery.

Case 2: Osteolytic lesion of multiple myeloma in weightbearing bones (e. g. axial skeleton) without surgery.

Case 3: Limited-stage Hodgkin lymphoma, Ann-Arbor stage II without risk factors after completion of 2 cycles of ABVD.

Case 4,5: Diffuse large B-cell lymphoma with initial abdominal bulky disease after completion of 6 cycles of R-CHOP.

4) With no information on PET-status

5) PET-positive after treatment

Case 6: Early stage indolent lymphoma in noncritical location.

1. *In which cases would you postpone the beginning of RT and how long? Multiple answers possible.*
2. *In which cases would you omit RT if there is a critical shortage of resources?*

1. *In which cases would you perform hypofractionation different from your usual treatment schedule? Which fractionation? Multiple answers possible.*
2. *In which cases would you consider a shortened RT treatment with dose reduction different from your usual treatment schedule? Which doses would you consider Multiple answers possible.*

*11. If a patient becomes COVID-19 positive before the start of RT, would you postpone the start of RT until receiving a negative test result? For which patient would you consider this approach and how long would you postpone treatment? Multiple answers possible.*

1. *If a patient becomes COVID-19 positive during RT series, would you interrupt treatment until receiving a negative test result? For which patients would you consider this approach? Multiple answers possible.*

**Second phase of the pandemic**

In the second phase of the pandemic treatment facilities are overwhelmed due to the exponential increase of patients. Consequently, this phase may be marked by a significant triage and prioritization.

1. *Do you consider reducing the number of hemato-oncological patients in your radiation oncology department?*

- Yes
- No

1. *Would you apply a risk-adapted triage before the start of RT in case of critical shortage of resources in your department? Which parameters do you consider relevant? Multiple answers possible*.

- Curative vs. palliative treatment
- Out-patient vs. in-patient
- Onset of symptoms/disease biology (aggressive vs. indolent)
- Alternative (systemic) therapies
- Patient’s immune status
- Immunosuppression by therapy
- Patient’s risk profile (age, smoking, hypertension, diabetes)
- No triage

1. *Would you initiate emergency radiation despite critically limited resources (within 24h)? In which scenarios?*

- Superior vena cava syndrome due to mediastinal tumor
- Intracranial pressure due to multifocal cerebral lymphoma
- Exacerbation of pain caused by vertebral involvement of multiple myeloma
- (Sub-)Acute cord compression with no surgical option
- (Sub-)Acute amaurosis due to orbital involvement
- No emergency RT

1. *Would you consider a chemotherapy-only conditioning regimen before allogenic stem cell transplantation in order to avoid time intensive radiation treatment like total-body irradiation (TBI)?*

- Yes
- No

**Clinical cases (question 17-20):**

1. *In which cases would you postpone the beginning of RT and how long? Multiple answers possible.*
2. *In which cases would you omit RT if there is a critical shortage of resources? Multiple answers possible.*
3. *Focusing on case 3 (early-stage Hodgkin lymphoma without risk factors): Would you consider a chemotherapy-only treatment regimen?*

- Yes
- No

1. *Rank/Prioritize all cases provided with consecutive numbers (1 being the most urgent treatment, 6 being the least urgent).*
